# Enhancing Discretion and Consistency in Emergency Contraception Counselling: Implementation of a Digital Support Tool in Community Pharmacies

**DOI:** 10.3390/pharmacy14030083

**Published:** 2026-06-09

**Authors:** Esther Spinatsch, Sabrina Zelger, Samuel S. Allemann

**Affiliations:** Pharmaceutical Care Research Group, Department of Pharmaceutical Sciences, University of Basel, 4056 Basel, Switzerlands.allemann@unibas.ch (S.S.A.)

**Keywords:** emergency contraception, community pharmacy, pharmacist-led counselling, digital health, digital counselling support tool, implementation study

## Abstract

Emergency contraception (EC) is a time-sensitive pharmacy service in which consistent, patient-centred counselling is important but can be challenging to deliver in routine practice. This prospective implementation study evaluated the feasibility and real-world use of a digital counselling support tool for pharmacist-led EC consultations in Swiss community pharmacies. The tool combines patient self-reporting with structured, evidence-based support for pharmacist counselling. All EC consultations between 1 November 2023 and 14 February 2026 using the tool in 10 Swiss community pharmacies were analysed descriptively (*n* = 3428), alongside a voluntary anonymous post-consultation survey (*n* = 148) assessing patient-reported experience and acceptance. Median total consultation duration was 11:32 min, including 4:27 min of direct pharmacist counselling, indicating partial transfer of assessment to the digital pre-consultation phase. Ulipristal acetate was dispensed in 71% and levonorgestrel in 26% of consultations. Prior to counselling, 80% of patients reported uncertainty regarding the optimal active ingredient, underscoring the relevance of pharmacist involvement. Survey respondents rated the tool as easy to use (97%), discreet (99%), and trustworthy (98%); 85% preferred it over standard paper-based procedures. These findings demonstrate the feasibility and patient acceptance of integrating a digital tool into routine EC services and suggest that such tools may support structured, evidence-based counselling within community pharmacy practice.

## 1. Introduction

Emergency contraception (EC) is a time-sensitive intervention and a key component of sexual and reproductive health. International guidance emphasises that timely access to EC is essential to prevent unintended pregnancy and reduce health inequities [1,2]. In many countries, community pharmacies play a central role in ensuring rapid access, either through pharmacist-led supply models or over-the-counter availability [3,4]. While simplified access may reduce delays, structured pharmacist counselling remains clinically relevant to ensure appropriate product selection, identify contraindications and drug interactions, address safeguarding concerns, and provide guidance on ongoing contraception and sexually transmitted infection (STI) prevention [5,6].

Switzerland represents a regulated pharmacist-led supply model in which oral EC, including levonorgestrel (LNG) and ulipristal acetate (UPA), is dispensed following a structured consultation supported by a national protocol [7,8]. EC is one of the most frequently provided pharmacy services. However, previous Swiss and European research has identified variability in counselling content and documentation quality [9,10,11]. Simulated patient studies have shown that key elements—such as STI risk assessment—are not consistently addressed [9], and qualitative research indicates that the consultation process may be perceived as uncomfortable or insufficiently discreet by some users [6,10]. In the context of EC counselling, discretion refers to the possibility of communicating sensitive sexual health information in a private, confidential, and non-judgemental manner. These findings highlight an inherent tension between structured quality assurance and the need for sensitive, patient-centred communication in time-pressured pharmacy settings.

In parallel, digital health tools are increasingly being integrated into healthcare workflows. Clinical decision support systems have demonstrated potential to improve consistency, reduce omissions, and support evidence-based decision-making; however, their impact depends strongly on usability, workflow integration, and user trust [12,13]. In the field of sexual and reproductive health, digital counselling and decision-support tools have shown promising results. Studies evaluating contraceptive decision aids and digital support systems report improved shared decision-making, increased knowledge, and positive provider perceptions [14,15,16]. Furthermore, research on sexual and reproductive health mobile applications highlights the importance of confidentiality, data security, community engagement, and perceived health benefits for user acceptance, particularly in sensitive contexts [17]. These findings suggest that digital self-assessment and counselling tools may offer specific advantages in consultations addressing intimate health concerns, as they can facilitate discreet disclosure of personal information and potentially reduce embarrassment. At the same time, evidence from Swiss and European pharmacy-based EC services demonstrates variability in counselling experiences and documentation quality [6,9,10,11], indicating scope for more structured support. However, highly structured digital tools may also influence the dynamics of sensitive consultations if not carefully integrated into routine practice. Although digital tools in reproductive health are increasingly used, real-world evidence on structured digital systems integrating patient input with support for pharmacist-led counselling in routine community pharmacy practice remains limited, particularly in emergency contraception.

The present study addresses this evidence gap by evaluating a digital counselling support tool implemented in Swiss community pharmacies for EC consultations. The tool combines patient self-reporting with structured support for pharmacist–patient interactions and documentation, aiming to enhance discretion, consistency of care, and evidence-based practice while preserving personalised, face-to-face counselling.

This prospective implementation study therefore aimed to evaluate the feasibility and real-world utilisation of a digital counselling support tool in routine EC practice, as well as patient-reported experience and acceptance. By linking large-scale consultation data with patient-reported outcomes, the study provides real-world evidence on the integration of structured digital support within community pharmacy-based reproductive health services.

## 2. Materials and Methods

### 2.1. Study Design

This study was designed as a prospective implementation study conducted in selected Swiss community pharmacies under routine practice conditions, with an embedded voluntary post-consultation cross-sectional patient survey. The primary objective was to evaluate the feasibility and real-world use of a digital counselling support tool (pharMe^®^, University of Basel, Basel, Switzerland) during EC counselling and to assess patient-reported experience and acceptance.

The study consisted of two components:

- A prospective collection of EC consultation data using the digital tool in participating pharmacies.
- An anonymous voluntary post-consultation online survey assessing patient experience.

Data from all EC consultations conducted using the tool between 1 November 2023 and 14 February 2026 were included in this analysis. The online survey was launched on 9 February 2024, and responses received up to 14 February 2026 were included in the present analysis.

### 2.2. Setting and Implementation

The study was conducted in 10 community pharmacies in the German- and French-speaking regions of Switzerland. Five participating pharmacies had previously collaborated with the study team in earlier research projects involving a predecessor version of the digital tool. Two additional pharmacies were actively approached by the research team due to their extended opening hours and high expected volume of EC consultations. Three further pharmacies became aware of the project through professional contacts and contacted the study team on their own initiative. All pharmacies expressing interest in participation received access to the tool, and all pharmacies that used the tool during the study period were included in the study. The tool was not commercially available during the study period.

The web-based digital counselling support tool has been designed to facilitate structured EC counselling. Patients complete a standardised questionnaire discreetly on their own smartphone prior to the consultation and transfer the data to the pharmacy to serve as the basis for counselling. If self-completion is not desired or feasible, the questionnaire can be completed by the pharmacist using a tablet or computer at the pharmacy. The tool collects clinically relevant information, including age, timing of unprotected intercourse, menstrual cycle characteristics, reason for EC request, and additional parameters relevant to EC selection. After data transmission, the tool guides the pharmacist through structured, evidence-based counselling supported by visual aids such as graphs and images for patient communication. At the end of the consultation, the digital tool generates an individualised patient information leaflet summarising key EC information and providing links to further sexual health resources. The clinical content and counselling pathways integrated into the tool were developed based on the current Swiss emergency contraception guidelines issued by the Interprofessional Expert Group for Emergency Contraception (IENK), which develops and publishes national EC guidance in Switzerland [7,8].

**Figure 1:**
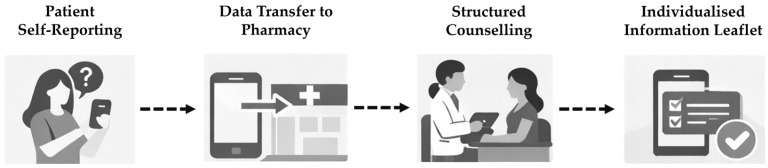
Schematic overview of the EC consultation process in community pharmacy practice using the digital counselling support tool. Figure created using ChatGPT (OpenAI, GPT-4/5), based on author-defined content.

The overview of the EC consultation process using the digital tool is schematically illustrated in Figure 1. The tool is intended to support high-quality, structured pharmacist–patient discussions tailored to the individual situation, without replacing face-to-face counselling.

### 2.3. Participants

The consultation dataset comprised all EC consultations recorded within the digital tool in participating pharmacies during the study period. Data were recorded in anonymised form, and no personally identifiable information was collected within the research dataset.

The survey dataset consisted of voluntary, anonymous online responses from patients who had used the digital tool during EC counselling. At the end of the consultation, a link to an online survey (Findmind Online Umfragen, Trogen AR, Switzerland) was displayed within the system and included in the digital patient information leaflet. No personally identifiable information was collected in the survey. Each response included a corresponding unique consultation identification number, which enabled linkage with the consultation dataset and identification of duplicate submissions. The study team had no access to any re-identification key, and the dataset was non-identifiable to the researchers. Inclusion criteria for survey analysis were the use of the digital tool pharMe^®^ during EC counselling and completion of at least one survey item.

### 2.4. Variables

The consultation dataset comprised routinely recorded variables collected through the digital tool during EC counselling. These included patient age, body mass index, reason for EC request, selected active ingredient, intake location, breastfeeding status, concomitant medication, previous EC use, EC use within the same cycle, and consultation duration derived from system timestamps.

A question on pre-consultation knowledge of the optimal active ingredient for the specific situation was additionally integrated into the digital tool at the end of the self-reporting, prior to pharmacist counselling. This variable was included to assess patients’ knowledge and decision certainty before counselling and to contextualise the relevance of pharmacist-led EC counselling.

The consultation dataset was analysed descriptively to summarise selected active ingredients, consultation duration, and pre-consultation knowledge regarding the optimal active ingredient.

The survey dataset included patient-reported measures on usability, perceived discretion, trust in the tool, data protection concerns, perceived impact on counselling, intention to reuse the digital tool, perceived knowledge gain and preference for written information. The full English translation of the survey questionnaire is provided in the Supplementary Materials (File S1). Although additional survey items were collected, the present analysis focused on variables relevant to implementation and patient-reported experience, specifically usability, discretion, trust, perceived impact on counselling, and intention to reuse the digital tool.

The survey questionnaire was reviewed by two pharmacists with expertise in pharmaceutical care research to assess structure, clarity, and relevance. Prior to implementation, it was pretested with 13 voluntary participants using their own smartphones to evaluate comprehensibility, logical flow, length, and technical functionality. The pretest did not result in major structural changes. The questionnaire was developed specifically for the present implementation study and was not formally psychometrically validated. The French version was independently reviewed for linguistic accuracy by a bilingual pharmacist familiar with the topic.

Likert-scale items were coded on a four-point ordinal scale (1 = strongly agree, 2 = agree, 3 = disagree, 4 = strongly disagree). For descriptive analysis, responses were collapsed into two categories: agreement (strongly agree and agree) and disagreement (disagree and strongly disagree). Responses marked “cannot say” were treated as missing values and excluded from percentage calculations.

### 2.5. Data Processing

Prior to analysis, predefined plausibility criteria were applied to the consultation dataset to identify implausible values likely resulting from input errors. Implausible entries for age (>70 years), height (<100 cm or >200 cm), and consultation duration (>59 min) were treated as missing values. Analyses were based on available data for each variable (available-case analysis), with no imputation of missing data.

Consultations that could not be unequivocally assigned to a participating pharmacy and consultations with a total recorded duration of less than one minute were excluded from analysis. In addition, test consultations were identified based on the presence of the term “test” in the free-text comment field and were removed prior to analysis.

For the survey dataset, responses without a corresponding EC consultation in the digital tool dataset and duplicate submissions for a single consultation were excluded. Matching between datasets was performed using a unique consultation identification number. Partially completed surveys were retained for analysis.

### 2.6. Statistical Analysis

Statistical analyses were performed using Microsoft^®^ Excel^®^ for Microsoft 365 MSO (Version 2602 Build 16.0.19725.20014), 64-bit.

Descriptive statistics were used to summarise consultation and survey data. Continuous variables were reported as medians and interquartile ranges (IQR). Categorical variables were presented as absolute frequencies and percentages.

No inferential statistical tests were performed. All analyses were descriptive in nature.

### 2.7. Ethical Considerations

The project was submitted for clarification of responsibility to the Ethikkommission Nordwest- und Zentralschweiz (EKNZ; reference number Req-2024-00129). The EKNZ determined that the project does not fall within the scope of the Swiss Human Research Act (Art. 2 para. 1), as it does not constitute research on diseases or on the structure and function of the human body as defined by the Act and therefore does not require formal ethical approval.

All data were collected and analysed in a fully anonymised form. No personally identifiable information was available to the study team.

### 2.8. Data Availability

The anonymised datasets generated and analysed during the current study are available from the corresponding author upon reasonable request. Data sharing is subject to data protection regulations and institutional policies. No personally identifiable information is contained within the research dataset.

### 2.9. Use of Generative Artificial Intelligence

Generative artificial intelligence tools were used to assist in structuring and drafting the manuscript text, for language editing and refinement, for translation of the survey questionnaire from German into English, and for the generation of Figure 1. No AI tools were used in data generation, data processing, statistical analysis, or interpretation of results.

## 3. Results

### 3.1. Consultation Dataset

#### 3.1.1. Study Population and Data Cleaning

A total of 3487 EC consultations were recorded using the digital tool during the study period across the 10 participating pharmacies. After data cleaning, 3428 consultations remained for final analysis (98%). A total of 59 consultations were excluded based on predefined criteria, including consultations with a total recorded duration of less than one minute (*n* = 6), test entries identified via the keyword “test” (*n* = 26), and consultations that could not be unequivocally assigned to a participating pharmacy (*n* = 27). Eight participating pharmacies were located in the German-speaking region of Switzerland and two in the French-speaking region. The number of consultations per pharmacy ranged from 6 to 1684 (median 129).

#### 3.1.2. Demographic, Consultation and Clinical Characteristics

Demographic, consultation and clinical characteristics of the consultation dataset and the survey population are summarised in Table 1.

#### 3.1.3. Pre-Consultation Knowledge of the Optimal Active Ingredient

Information on patient-reported pre-consultation knowledge of the optimal active ingredient was available for 3266 consultations. Overall, 667 patients (20%) reported believing they knew which active ingredient was optimal prior to counselling, whereas 2599 patients (80%) reported uncertainty.

Among those reporting prior knowledge, 250 patients (8% of total consultations) indicated LNG as their presumed optimal choice; of these, 172 (69%) received LNG. A total of 417 patients (13% of total consultations) indicated UPA; of these, 334 (80%) received UPA.

### 3.2. Satisfaction Survey Population

#### Survey Population and Data Cleaning

A total of 233 survey responses were received during the study period across the 10 participating pharmacies. After excluding 11 unmatched records, 2 duplicate submissions, and 72 completely empty questionnaires, 148 responses were retained for final analysis. This corresponds to a response rate of 4.5%, based on 148 responses from 3312 consultations conducted during the survey period. The estimated margin of error for proportions was approximately ±8% at a 95% confidence level; however, as participation was voluntary, the results may be subject to selection bias.

Of the analysed surveys, 128 (86.5%) were completed in German and 20 (13.5%) in French. Responses were unevenly distributed across pharmacies, ranging from 1 to 69 surveys per site. Nearly half of all responses originated from one pharmacy (69/148; 46.6%), followed by two sites contributing 39 (26.4%) and 20 (13.5%) responses, respectively. The remaining pharmacies each contributed between 1 and 12 responses.

### 3.3. Demographic, Consultation and Clinical Characteristics of the Survey Population

Demographic, consultation and clinical characteristics of the survey population, as well as those of the consultation dataset, are summarised in Table 1.

Information on the highest educational attainment was available for 83 participants. Among these, 1 (1%) reported no completed school qualification, 8 (10%) reported completion of compulsory schooling, 30 (36%) reported completion of upper secondary education, and 44 (53%) reported tertiary education.

### 3.4. Patient-Reported Experience with the Digital Counselling Tool

Overall, participants reported high usability, trust, and discretion when using the digital tool, with minimal perceived disruption to the counselling process. Agreement (strongly agree/agree) and disagreement (disagree/strongly disagree) with selected survey items across the domains of usability, discretion and trust, and perceived impact on counselling are presented in Table 2.

### 3.5. Intention to Reuse the Digital Counselling Tool

Ninety-six participants answered the question on whether they would use the digital tool again if EC was needed or prefer the standard paper-based procedure. A total of 82 participants (85%) indicated a preference for the digital tool over the standard paper-based procedure. Thirteen participants (14%) reported no preference between the two approaches, and one participant (1%) stated that they would prefer the standard paper-based procedure.

## 4. Discussion

### 4.1. Principal Findings

This prospective implementation study demonstrates that the digital counselling support tool can be integrated into routine EC services in Swiss community pharmacies with high feasibility and strong patient acceptance. A total of 3428 EC consultations were documented across 10 pharmacies in both German- and French-speaking regions of Switzerland. The participating sites were structurally heterogeneous, including centrally located urban pharmacies with extended opening hours as well as smaller neighbourhood pharmacies, which is reflected in the marked variation in consultation numbers. In the context of approximately 100,000 EC supplies annually in Switzerland [18], this represents a meaningful real-world segment of national service provision.

The median duration of direct pharmacist counselling was approximately 4:30 min within a total consultation time of around 11:30 min. More than half of the total duration occurred prior to the face-to-face interaction, including patient self-reporting and waiting time. These findings suggest that structured digital self-reporting may allow part of the information collection process to be shifted to the digital pre-consultation phase, enabling a more focused face-to-face interaction between the pharmacist and patient.

Overall acceptance of the digital tool was high. The vast majority of respondents rated the tool as easy to use (97%), reported being able to document their concern discreetly (99%), and considered it trustworthy (98%). Only a small proportion indicated that the use of the tool on a tablet or computer disturbed the counselling conversation (11%), while most participants confirmed that they were able to ask questions before, during, or after the consultation (94%). These findings suggest that digital self-reporting was perceived as a supportive rather than disruptive component of the consultation process.

### 4.2. Results in the Context of Existing Evidence

The characteristics of both the overall consultation dataset and the survey population were broadly comparable to those observed in previous Swiss studies with regard to age, BMI, and key clinical parameters [6,19], suggesting reasonable representativeness of routine EC users. However, longer consultation times in the survey subgroup may indicate higher engagement among respondents and should be interpreted cautiously as a potential participation bias. In addition, while the consultation dataset included a broadly balanced distribution of consultations across German- and French-speaking regions, despite differences in the number of participating pharmacies, survey responses were predominantly obtained from patients who had consulted German-speaking pharmacies. This imbalance suggests a potential regional response bias and may limit the generalisability of patient-reported outcomes across linguistic regions.

The integration of a pre-consultation question assessing patients’ knowledge of the optimal active ingredient provides insight into the ongoing debate on over-the-counter (OTC) access to EC. The World Health Organization has highlighted that, despite more than two decades of OTC availability in many countries, published evidence on women’s knowledge, correct use, and information needs remains limited across different settings and has explicitly called for further research to improve understanding [4]. Recent economic evidence further nuances the debate on OTC access. A within-country analysis reported that OTC availability of EC substantially increased sales but had no effect on abortions or STIs, while fertility rates increased, and substitution from regular hormonal contraception to EC was observed [20]. These findings suggest that easier access alone does not necessarily improve reproductive health outcomes.

In this context, the present study’s finding that 80% of patients were uncertain about the optimal active ingredient prior to counselling underscores the continued relevance of structured, evidence-based assessment within pharmacy-based EC provision, as practised in the Swiss model.

Previous Swiss survey data indicate that EC counselling is generally perceived as reassuring and beneficial [6], while also identifying variability in counselling practices and concerns about judgement and discretion that may deter some women from seeking care. Similarly, qualitative research from Australia has highlighted challenges in the practical use of EC practice guidelines, with pharmacists reporting limited confidence, knowledge gaps, and difficulties in interpreting guideline recommendations [21]. Together, these findings suggest that the existence of guidelines alone does not ensure consistent implementation in routine practice. Structured digital support tools may help translate guideline recommendations into practical, workflow-integrated decision support. In this context, pharMe^®^ was developed to support structured and discreet EC consultations by embedding guideline-based assessment directly into routine pharmacy workflows.

Nevertheless, 27% of respondents considered the counselling conversation unnecessary, a proportion comparable to the 25% previously reported in Switzerland [6]. This suggests that scepticism towards counselling is not specific to the implementation of a digital tool but reflects a broader perception among a subset of EC users. Closer examination, however, reveals a discrepancy between perceived and actual informational needs: among those who considered counselling unnecessary, more than 90% (30/33) had indicated uncertainty regarding the optimal active ingredient.

These findings point to two distinct components of EC counselling: the structured clinical assessment required for safe and appropriate product selection, and broader communicative and sexual health counselling elements.

### 4.3. Clinical Implications

While structured clinical assessment should remain standard practice, the scope of additional counselling may be adapted to individual preferences. Digital self-reporting tools such as pharMe^®^ can support this differentiation by capturing counselling preferences, streamlining data collection, and enabling more focused, tailored pharmacist–patient interactions.

In the context of increasing service demands and workforce constraints in primary care, such digitally supported workflow integration represents a pragmatic strategy to support structured EC provision while preserving professional oversight. By addressing previously reported concerns regarding variability in counselling quality, perceived judgement, and lack of discretion, the present findings further suggest that structured digital integration can enhance perceived privacy and support structured evidence-based counselling within pharmacy-based EC services.

### 4.4. Strengths and Limitations

This study has several strengths, including its prospective implementation under routine practice conditions and the large real-world consultation dataset collected over a period of more than two years. The inclusion of structurally heterogeneous pharmacies from both German- and French-speaking regions enhances ecological validity. Linking consultation data with patient-reported outcomes further enables a combined assessment of workflow feasibility and user experience.

However, we acknowledge several limitations. The survey component is subject to potential selection bias due to voluntary participation and a low response rate, resulting in a margin of error of approximately 8% at a 95% confidence level. In addition, survey responses were predominantly obtained from patients who had consulted German-speaking pharmacies, despite a broadly balanced distribution of consultations across German- and French-speaking regions. This imbalance indicates a potential regional response bias and may further limit the generalisability of patient-reported outcomes across linguistic settings.

Furthermore, the study relied on non-probabilistic sampling at the pharmacy level, which may have led to the inclusion of more motivated pharmacies with a particular interest in counselling quality. Several participating pharmacies had previous collaborations with the study team involving an earlier version of the digital tool or contacted the study team on their own initiative after becoming aware of the project through professional networks. This may have increased the likelihood of including pharmacies with above-average interest in digital innovation and structured counselling services. The applicability of the findings to pharmacies with lower levels of digital readiness may therefore be limited. Together with the limited number of participating pharmacies and the predominance of German-speaking sites, this may restrict generalisability. In addition, only pharmacies from the German- and French-speaking regions of Switzerland were included for linguistic reasons.

The analyses were purely descriptive, and no control group was included; therefore, causal conclusions regarding the comparative effectiveness of digital versus conventional counselling cannot be drawn. Long-term clinical or behavioural outcomes were not assessed. Accordingly, the present study does not allow conclusions regarding the clinical impact of the digital tool, including pregnancy outcomes.

### 4.5. Future Research

Future research should evaluate the implementation of the digital tool within a broader network of community pharmacies to assess scalability and generalisability across different organisational contexts. Active communication strategies could be explored to inform patients about the availability of digital self-reporting and to enable informed choice between digital and conventional counselling modalities.

In addition, future iterations of the tool could incorporate an item assessing individual counselling preferences, allowing consultations to be more closely aligned with patients’ expectations. Following such adaptations, a renewed evaluation of patient satisfaction and consultation dynamics would be warranted to assess the impact of preference-based differentiation on acceptance, workflow, and perceived quality of care.

## Figures and Tables

**Table 1:** Demographic, consultation and clinical characteristics of the consultation dataset and the survey population.

Characteristics	Consultation Dataset	Survey Population
Age [years], mean (SD)	26 (7.4)	26 (7.8)
BMI [kg/m^2^], mean (SD)	22.1 (4.2)	22.1 (4.6)
Time to counselling start [minutes], median (IQR)	6:17 (5:00)	8:04 (6:03)
Pharmacist counselling duration [minutes], median (IQR)	4:27 (4:33)	6:33 (5:24)
Total consultation duration [minutes], median (IQR)	11:32 (7:52)	15:28 (9:47)
	*n* (%)	*n* (%)
Pharmacy region	*n* = 3428	*n* = 148
German-speaking region	1738 (51%)	128 (86%)
French-speaking region	1690 (49%)	20 (14%)
Reason for EC request	*n* = 3388	*n* = 145
Condom failure	1703 (50%)	104 (72%)
No contraception used	1429 (42%)	34 (23%)
Hormonal contraception issue	185 (5%)	3 (2%)
Other situation	71 (2%)	4 (3%)
Active ingredient dispensed	*n* = 3039	*n* = 140
Ulipristal acetate	2152 (71%)	115 (82%)
Levonorgestrel	782 (26%)	21 (15%)
Double Dose Levonorgestrel	31 (1%)	3 (2%)
No product dispensed	74 (2%)	1 (1%)
Taken on-site (pharmacy)	*n* = 2759	*n* = 134
Yes	2662 (96%)	133 (99%)
No	97 (4%)	1 (1%)
Breastfeeding	*n* = 3394	*n* = 148
Yes	47 (1%)	3 (2%)
No	3347 (99%)	145 (98)
Concomitant medication	*n* = 3402	*n* = 148
Yes	632 (19%)	29 (20%)
No	2770 (81%)	119 (80%)
Previous EC use (lifetime)	*n* = 3400	*n* = 148
Yes	2252 (66%)	95 (64%)
No	1148 (34%)	53 (36%)
EC use within the same cycle	*n* = 2249	*n* = 95
Yes	118 (5%)	7 (7%)
No	2131 (95%)	88 (93%)

**Table 2:** Patient-reported experience with the digital tool among survey participants.

	Item	Agreement *n* (%)	Disagreement *n* (%)	*n* (Valid)
Usability and Interaction Preference	pharMe was easy to use	142 (97%)	5 (3%)	147
	I had difficulties completing the questions on my own	20 (14%)	126 (86%)	146
	I would have preferred answering the questions orally	30 (23%)	103 (77%)	133
Discretion and Trust	I was able to document my concern discreetly	143 (99%)	2 (1%)	145
	pharMe appeared trustworthy	141 (98%)	3 (2%)	144
	I have concerns regarding data protection	26 (17%)	126 (83%)	152
Perceived Impact on Counselling	The use of pharMe on a tablet or computer disturbed the counselling conversation	13 (11%)	108 (89%)	121
	I was able to ask questions before, during or after the consultation	115 (94%)	7 (6%)	122
	The counselling conversation was unnecessary	33 (27%)	88 (73%)	121

## Data Availability

The anonymised datasets generated and analysed during the current study are available from the corresponding author upon request. No personally identifiable information is contained in the research dataset.

## Supplementary Materials

The following supporting information can be downloaded at: https://www.mdpi.com/article/10.3390/pharmacy14030083/s1, File S1: Full English translation of the patient survey questionnaire used in this study.

## Abbreviations

The following abbreviations are used in this manuscript:

BMI	Body mass index
EC	Emergency contraception
EKNZ	Ethikkommission Nordwest- und Zentralschweiz (Ethics Committee of Northwestern and Central Switzerland)
IQR	Interquartile range
LNG	Levonorgestrel
SD	Standard deviation
STI	Sexually transmitted infection
UPA	Ulipristal acetate
