# Supplementary material for: Enhancing Discretion and Consistency in Emergency Contraception Counselling: Implementation of a Digital Support Tool in Community Pharmacies
Source: Pharmacy (Basel). 2026 Jun 9;14(3):83. doi: 10.3390/pharmacy14030083 (PMC13307340; doi:10.3390/pharmacy14030083)
Supplement: Supplementary file 1 [file pharmacy-14-00083-s001.zip › pharmacy-4308198-supplementary.pdf]

Supplementary Material – File S1
Full English Translation of the Patient Survey Questionnaire

1. **You were able to answer personal questions directly on your own smartphone using pharMe. What is your opinion on the following statements?**

Options: Strongly agree | Tend to agree | Tend to disagree | Strongly disagree | Cannot say

a) pharMe is easy to use.
b) I would have preferred to answer the questions orally rather than on my smartphone.
c) I was able to describe my concern discreetly using pharMe.
d) I found it difficult to answer the questions on my own.
e) pharMe appears trustworthy.
f) I am concerned about data protection.

2. **What is your opinion on the following statements regarding the counselling?**

Options: Strongly agree | Tend to agree | Tend to disagree | Strongly disagree | Cannot say

a) I found the counselling unnecessary.
b) The illustrations in pharMe were helpful in understanding the menstrual cycle and the mechanism of action of emergency contraception.
c) I had difficulties to talk about this intimate topic.
d) I was able to ask questions before, during, or after the counselling.
e) Using pharMe on a computer or tablet disrupted the counselling.

3. **The following topics may have been discussed during the counselling. Did you learn anything new?**

Options: Yes, learned something new | No, I already knew this | Topic was not discussed

a) Effect of oral emergency contraception
b) Side effects of oral emergency contraception
c) What to do if menstruation does not occur
d) Contraception in the days following use of oral emergency contraception
e) Contraceptive methods in general
f) Sexually transmitted infections
g) Further counselling services (for example, sexual health counselling centers)

4. **At the end of the consultation, did you receive an information leaflet via QR code?**

a) Yes
b) No

5. If Question 4 = YES: What is your opinion on the following statements?

Options: Strongly agree | Tend to agree | Tend to disagree | Strongly disagree | Cannot say

a) I intend to read the leaflet or have already read it.
b) I saved the link in order to read something again later if needed.
c) I would have preferred a printed leaflet (for example, a flyer).
d) I am glad to have received written information.
e) It is tiring to read the leaflet on a smartphone.
f) The information in the leaflet is easy to understand.

6. If question 4 = NO: Which of the following applies to you?

a) I would have appreciated receiving a leaflet.
b) I do not wish to receive written information.

7. If you needed emergency contraception again, would you want to use pharMe again?

a) Yes
b) No
c) No preference

8. What is your highest level of education?

a) No school qualification yet
b) Compulsory schooling completed
c) Upper secondary level completed (e.g. apprenticeship, secondary school)
d) Tertiary level completed (e.g. technical college, university)
e) Prefer not to say

9. Is there anything else you would like to tell us?
